# An Investigation of Meat Eating in Samples from Australia and Germany: The Role of Justifications, Perceptions, and Empathy

**DOI:** 10.3390/ani14020211

**Published:** 2024-01-09

**Authors:** Katherine Northrope, Tiffani Howell, Emiko S. Kashima, Benjamin Buttlar, Gudrun Sproesser, Matthew B. Ruby

**Affiliations:** 1 School of Psychology and Public Health, La Trobe University, Bendigo 3552, Australia; t.howell@latrobe.edu.au; 2 School of Psychology and Public Health, La Trobe University, Melbourne 3086, Australia; e.kashima@latrobe.edu.au (E.S.K.); m.ruby@latrobe.edu.au (M.B.R.); 3 Department of Psychology, University of Trier, 54296 Trier, Germany; buttlar@uni-trier.de; 4 Department of Health Psychology, Johannes Kepler University Linz, 4040 Linz, Austria; gudrun.sproesser@jku.at

**Keywords:** animals, culture, empathy, meat, taste, vegetarianism

## Abstract

**Simple Summary:**

Despite concerns about animal welfare, and health and environmental issues associated with eating meat, meat consumption continues to increase in many countries around the world. The aim of this pre-registered study was to identify predictors of meat consumption among 399 Germans and 399 Australians. Participants reported current and intended meat consumption, reasons for eating meat, attitudes towards animals, and perceptions of animal farming. In both countries, the more people enjoyed the taste of meat, the more often they ate it, and the more empathy they had towards farmed animals, the less often they ate it. People who thought they would find it easy to reduce meat consumption had greater intentions to do so. Campaigns to reduce meat consumption should focus on increasing empathy towards farmed animals, highlighting tasty plant-based alternatives and outlining ways to make the transition easier.

**Abstract:**

Despite concerns about animal welfare, and health and environmental issues associated with eating meat, meat consumption has continued to increase worldwide, including in Australia. One exception to this is Germany, with 2021 meat consumption levels being the lowest in the last 30 years. This pre-registered study investigated socio-cultural variables associated with meat consumption in Germany (*n* = 399) and Australia (*n* = 399) in a cross-sectional online survey. Participants reported levels of current and intended meat consumption, and they completed measures of speciesism, motivations to eat meat, empathy, animal farming perceptions, perceived behavioural control (PBC) over meat eating, and avoidance and dissociation regarding the animal origins of meat. In both Australia and Germany, enjoying the taste of meat positively predicted consumption and empathy towards farmed animals negatively predicted consumption. PBC was a strong positive predictor of intentions to reduce meat consumption in both countries. Empathy and liking the taste of meat were among the best predictors of red meat and poultry consumption, suggesting that interventions to reduce meat consumption may work best by targeting these factors while also increasing people’s sense of control over their food choices.

## 1. Introduction

Reducing meat intake is beneficial for health, the environment, and animal welfare [1,2,3,4]. Around 80 billion farmed animals are killed for meat per year worldwide [5]. Estimates of global greenhouse gas emissions from the animal agriculture industry range from 12 to 18%, and animal agriculture is seen as one of the biggest contributors to climate change [6]. Agriculture production also heavily contributes to deforestation rates and biodiversity loss and uses large amounts of freshwater [7]. For these reasons, calls have been Increasing for people to eat less meat, or stop eating it altogether, to reduce impacts on the environment and mitigate the risk of further climate change [8,9,10]. Furthermore, red meat has been associated with several cancers as well as cardiovascular and metabolic diseases and has been listed as a probable carcinogen, while processed meat has been declared carcinogenic [10]. There are also growing concerns about zoonotic diseases and antibiotic-resistant bacteria arising from meat production [11].

Despite all this, meat consumption worldwide has been increasing [12]. A prime example of this is Australia, where meat consumption has increased by 18% in the last 20 years [13]. A poll by the ABC found that only 3% of Australia consider themselves vegetarian [14]. Contrast this with meat consumption in Germany, which appears to be declining, with 2021 levels being the lowest in the last 30 years [15]. Estimates suggest the prevalence of vegetarians and vegans in Germany ranges from 4.3% to 12% [16,17] as of 2021. Against this background, the present cross-cultural study examines how a range of social–cognitive variables are related to current meat consumption and intentions to reduce meat consumption in the future. Specifically, we investigate justifications for and perceived behavioural control about eating meat, speciesism, empathy, and animal farming perceptions.

### 1.1. Justifications for Eating Meat

Joy [18] argued that most people justify meat consumption through the Three Ns: the beliefs that eating meat is normal (eating meat is a commonly accepted behaviour), natural (humans evolved to eat meat), and necessary (humans need meat to be healthy). Research by Piazza et al. [19] built on Joy’s work by adding a fourth N, nice (people enjoy eating meat). The 4Ns capture most justifications for eating meat, and omnivores tend to endorse the 4Ns more than vegetarians and vegans [19]. The 4Ns help relieve omnivores from cognitive dissonance by denying personal responsibility for any harm inflicted on animals farmed for food [20], and those who endorse the 4Ns more experience less guilt about eating meat [19]. The Motivations to Eat Meat Inventory (MEMI) was later developed to measure the 4Ns and distinguish motivations for each of the 4N subscales separately rather than them loading onto a single factor of overall meat justification [21].

Other research by Rothgerber [22] investigated different meat-eating justification (MEJ) strategies. Direct strategies included pro-meat (e.g., “I enjoy eating meat too much to ever give it up”); denial (e.g., “Animals don’t really suffer when being raised and killed for meat”); and hierarchical justification (e.g., “Humans are at the top of the food chain and meant to eat animals”). Indirect strategies were avoidance (e.g., “I try not to think about what goes on in slaughterhouses”) and dissociation (e.g., “When I look at meat, I try hard not to connect it with an animal”). Compared to women, men tended to endorse meat eating more and more frequently used direct strategies [22]. This may in part explain why they also reported eating meat more than women. Women, on the other hand, endorsed indirect strategies more than men. The direct strategies of the MEJ are significantly correlated with the overall endorsement of the 4Ns [19]. However, the MEJ’s indirect strategies are not significantly correlated with the 4Ns, as they are more passive, apologetic strategies [19].

### 1.2. Speciesism and Empathy

Speciesism, originally discussed in philosophy, is an ideology that prioritizes the interests of one’s own species (i.e., *homo sapiens*) over other species and that assigns moral worth based on species group membership [23,24]. Speciesism has been compared to other forms of discrimination in human societies such as racism and sexism [25]. Caviola et al. [23] developed a speciesism scale and, across several studies, showed that speciesism was associated with other forms of prejudice (e.g., racism, sexism, homophobia) as well as dietary choices. Other research has shown that vegetarians and vegans tend to endorse speciesist attitudes less than omnivores [26].

Empathy refers to understanding and relating to another individual’s experience on a cognitive and emotional level [27]. Although empathy towards animals and empathy towards humans are two separate constructs [28], higher levels of empathy towards humans have been associated with more positive attitudes towards animal welfare [29]. Research suggests that vegetarians tend to be more empathetic towards both humans and other animals than omnivores [30,31,32,33]. Childhood pet ownership has been associated with higher levels of empathy towards animals [28], both of which have been associated with higher levels of meat avoidance [34].

### 1.3. Perceived Behavioural Control

The Theory of Planned Behaviour (TPB) has commonly been used in research examining intentions to reduce meat consumption [35,36]. TPB states that a person’s intended behaviour can be predicted by their attitude towards the behaviour, subjective norms (what they believe important others think about the behaviour), and perceived behavioural control (their ability to perform the behaviour) [37]. The 4Ns scale [19] arguably taps into attitudes towards animals and subjective norms, and attitudes are also captured via speciesism. In addition, perceived behavioural control was significantly linked to consumers’ willingness to adopt a more plant-based diet [35,36].

### 1.4. Animal Farming Perceptions

Animal welfare concerns about animals raised for meat, such as cows, chickens, and pigs, include issues related to these animals living in crowded, confined conditions; painful procedures such as castration and beak trimming without pain relief; and risks associated with transportation and slaughter practices [38]. Consumers find different ways to cope with unpleasant information about where their meat comes from. Vegetarians and participants who ate meat less than three times a week reported being less willing to ignore negative information about animal farming practices, whereas other consumers preferred to be “strategically ignorant” by actively ignoring negative information about animal farming practices [39]. Research in the European Union found that those who claimed to know more about animal farming conditions were more likely to see animal welfare as being important [40].

While these studies suggest that having more animal welfare knowledge may lead to more ethically informed decision making, much previous research has solely relied on participants’ self-assessment of their level of knowledge. Research in Australia has found that consumers’ perceived knowledge of farming practices was only weakly correlated with more objective measures of their knowledge [41]. People tend to overestimate their knowledge of farming practices, and there are many misconceptions that exist, suggesting a need for more objective measures of knowledge to help understand what consumers know about where their meat comes from [42].

The general Australian population appears to have limited knowledge of animal agricultural practices [43]. Nonetheless, research into Australians’ opinions indicates that most consider animal welfare to be important, disagree that the demand for food is more important than humane treatment [44], and think that animal welfare conditions should be improved [45]. Men in Australia tend to be less concerned than women about animal welfare and environmental issues related to meat production [43]. Between 2013 and 2019, consumer concerns about farmed animal welfare have increased, and trust in land and sea transport of livestock has decreased, which may be linked to campaigns by organizations such as Animals Australia [46].

Qualitative research found that German meat consumers tended to have less specific knowledge about modern poultry production than veg*ans (vegetarians and vegans) [47], although scepticism of production systems was found in both groups. The decline in meat consumption in Germany has primarily been attributed to increasing awareness among Germans of intensive animal farming practices and their environmental impact [48]. Indeed, Kayser et al. [49] found that low-meat consumers in Germany were more concerned with animal welfare and environmental impacts than those with high meat consumption.

### 1.5. The Present Study

Given the increased concerns for animal welfare in both countries, it was unclear why meat consumption is increasing among Australians while decreasing among Germans. The present study thus examined predictors of meat consumption and intentions to reduce meat consumption to shed light on country-level differences in Australia vs. Germany. Given that gender differences have consistently been reported for meat consumption and its associated variables, we also investigated gender differences for these variables [22,50].

For this study, we were guided by the principles of Open Science [51], including collaboration, transparency, and reproducibility. We pre-registered the study’s materials, aims, and hypotheses with the Open Science Framework (https://osf.io/v74m5, accessed on 2 October 2022) prior to data collection.

**Hypothesis 1** **(H1).** *Meat consumption would be positively associated with speciesism, the four subscales of the MEMI, and the Avoidance and Dissociation subscales of the MEJ. We further predicted it would be negatively associated with empathy towards farmed animals, perceived frequency of common animal farming practices (e.g., separating newborn calves from their mothers and killing unwanted male chicks), and identifying as a woman.*

**Hypothesis 2** **(H2).** *Intentions to reduce meat consumption would be negatively associated with speciesism, the four subscales of the MEMI, and the Avoidance and Dissociation subscales of the MEJ. We further predicted it would be positively associated with empathy towards farm animals, perceived frequency of common animal farming practices, and perceived behavioural control.*

**Hypothesis 3** **(H3).** *Relative to men, women would score lower on speciesism, the MEMI, and current meat consumption, and they would score higher on animal farming perceptions, empathy towards farmed animals, and the Avoidance and Dissociation subscales of the MEJ. It is possible that there may be country-wide differences for the strength of these associations.*

## 2. Materials and Methods

### 2.1. Participants

We recruited participants through Prolific, an online participant recruitment platform that allows researchers to recruit participants easily and quickly [52], in September, 2022. Inclusion criteria were that participants were at least 18 years of age and (1) either lived in either Australia and spoke English or (2) lived in Germany and spoke German. According to G*Power, 390 participants were required to detect an effect size of *f*^2^ = 0.04 with 90% power (α = 0.05). We recruited 399 participants from Australia (196 men, 197 women, 5 non-binary/diverse, 1 preferred not to say; *M* age: 35.87, *SD*: 12.96, Range 18–85) and 399 from Germany (199 men, 192 women, 6 non-binary/diverse, 2 preferred not to say; *M* age: 31.18, *SD*: 9.97, Range 18–71). The majority of participants in both Australia (Bachelor’s degree 39.8%; post-graduate degree 24.4%) and Germany (Bachelor’s degree 28.3%; post-graduate degree 28.3%) had some form of university education. Information for dietary groups is presented in Table 1.

### 2.2. Materials

After completing the Participant Information and Consent Form, participants completed the following measures:

The Motivations to Eat Meat Inventory (MEMI). The MEMI [21] was used to measure each component of the 4N (i.e., Necessary, Normal, Natural, and Nice). Participants were presented with a list of 19 reasons to eat meat and other animal products like eggs and dairy and asked to rate each reason’s importance (e.g., “Eating meat is part of our biology”) on a 7-point scale ranging from 1 “least important” to 7 “most important” with 4 “moderately important” as a mid-point. Vegetarians and vegans were asked to answer based on the “reasons they might have to eat meat, even though they do not”. Internal reliability was excellent in both samples (Australia α = 0.91, Germany α = 0.93).

Meat Eating Justification (MEJ) Scale. To complement the direct motivations to eat meat captured in the MEMI, participants also completed the Avoidance and Dissociation subscales from Rothgerber’s [22] MEJ scale. There were originally three items for both Avoidance and Dissociation used [22]; however, due to an error in programming the survey, one Dissociation item was omitted. A sample item for the Avoidance scale is “I try not to think about what goes on in slaughterhouses” and a sample item for the Dissociation scale is “I do not like to think about where the meat I eat comes from”. Participants responded on a 7-point scale from 1—“Strongly disagree” to 7—“Strongly agree”. Higher scores indicated higher levels of Avoidance and Dissociation. These scales had lower reliability than the other measures but were still considered acceptable except Avoidance for Germany (Avoidance: Australia α = 0.73, Germany α = 0.60; Dissociation: Australia α = 0.79, Germany α = 0.83).

Animal Empathy. We used Kunst and Hohle’s [53] adaptation of the empathy subscale of the Interpersonal Reactivity Index (IRI) [54]. The original study, which attempted to manipulate participants’ empathy levels, used several different photos in experimental conditions that were designed to remind participants of the animal that died to produce the meat to different degrees to elicit more or less empathy. As we were not trying to influence participants’ levels of empathy, we provided participants with photos of processed chicken, pork, and beef. Participants responded on a 7-point Likert scale ranging from 1 “Totally disagree to 7 “Totally agree” to five statements such as “When I see the picture above, I feel sorry for the animal that was slaughtered”. Two items were reverse-scored. Higher scores indicated higher levels of empathy toward farm animals. Internal reliability was excellent in both samples (Australia α = 0.95, Germany α = 0.94).

Speciesism. Speciesism was assessed by Caviola et al.’s [23] Speciesism Scale. This scale consists of six questions, one of which is reverse-scored—e.g., “Morally, animals always count for less than humans”. Participants answered on a 7-point scale from 1—“Strongly disagree” to 7—“Strongly agree” with higher scores indicating greater endorsement of speciesist attitudes. Internal reliability was high in both samples (Australia α = 0.83, Germany α = 0.87).

Perceived Behavioural Control (PBC) was measured using three questions on a 7-point scale, which was adapted from a previous study investigating the Theory of Planned Behaviour in predicting intentions to eat healthier [55]. Higher scores indicated more perceived behavioural control. The item “How easy or difficult do you think it will be for you to eat less meat in the future?” was answered with a response scale ranging from 1 “Very Easy” to 7 “Very Difficult” and was reverse-scored. “How much control do you have over eating less meat in the future?” was answered with a response scale ranging from 1 “No Control” to 7 “Complete Control”. “If I want to, I can easily eat reduce my levels of meat consumption in the future” was answered with a response scale ranging from 1 “Strongly Disagree” to 7 “Strongly Agree”. After reverse scoring as needed, the scores for these three items were averaged; internal reliability was good in both samples (Australia α = 0.76, Germany α = 0.81).

Animal Farming Perceptions (AFP). We developed a measure of how frequently participants think common practices occur in their country based on previous work by Northrope and Ruby [56]. We carefully considered what practices to include for this study that were applicable in both countries, as Germany generally has better welfare standards for farmed animals than Australia [57]. For each practice, participants indicated “How often do you think the following happens in (Germany/Australia)?” on a slider from 0% of the time to 100%. After reverse-scoring three items that are probably not common practices (indicated in Table 2 with R), we averaged the items to form a composite. The items, their mean scores, and standard deviations are presented in Table 2. The overall reliability of this measure was lower than other measures used in this study (Australia α = 0.64, Germany α = 0.67). This is perhaps not surprising, as we are asking about practices that occur with different frequencies and which involve different species of animals.

Meat consumption and reduction intention. Participants indicated how many days per week they ate poultry, red meat, and fish/shellfish, selecting a number from 0 to 7. Participants then indicated “In the next six months, to what extent do you intend to reduce your meat consumption?” on a 7-point scale from “not at all” to “fully”. Participants also indicated which category best fit their current eating habits—omnivore, omnivore with a few restrictions, reducetarian, partial vegetarian, vegetarian, or vegan.

Participants also provided demographic information including gender, age, education, country of birth, and country of parents’ birth.

### 2.3. Procedure

The study was approved by the Human Research Ethics Committee of La Trobe University (HREC21204). Participants who met the inclusion criteria via the standard prolific pre-screeners and agreed to participate were given access to a link to complete the survey in QuestionPro. The median completion time was 7 min and participants were paid £1, which is above Prolific’s specified minimum rate of £6/h. Data were then analysed in IBM SPSS Statistics (version 28.0).

### 2.4. Data Analysis

We initially planned to test Hypothesis 1 and 2 using a multiple regression to assess the extent to which speciesism, AFP, empathy, the Normal subscale of the MEMI, and the Avoidance and Dissociation subscales of the MEJ predicted current levels of poultry intake, red meat intake, and fish consumption as well as PBC for intentions to reduce meat consumption by entering all variables simultaneously. However, after viewing the correlation between the variables above, Normal had the weakest relationship of the 4Ns with the dependent variables. Because of this, we decided to include all the 4Ns in our regression for completeness. The results for the original regression plan can be accessed at https://osf.io/j8vtp/, accessed on 22 August 2022. For Hypothesis 2, predicting intentions to reduce meat consumption, we excluded vegetarians and vegans from the analysis, as they already do not eat any meat. For Hypothesis 3 relating to gender comparisons, we excluded vegetarians and vegans from the analysis for the intentions to reduce comparison as well. As we did not explicitly outline this in our pre-registration, we also provide the results of these regressions and *t*-tests, which are largely the same, via the above OSF link.

For any analyses involving gender, given the small number of participants in the non-binary and gender diverse categories, we excluded these participants and instead ran gender as a binary variable with men and women only.

We assessed the assumptions of normality, linearity, homoscedasticity, and independence of residuals as well as the presence of outliers by examining the residuals scatterplots and found that none of the assumptions were violated. Where the assumption of the homogeneity of variance has been violated by the Levene’s Test for Equality of Variances, the results were taken from the result for equal variances not assumed.

## 3. Results

Descriptive statistics for meat consumption and intentions to reduce and the composite scores for the scales used in this study are reported in Table 3 for Australia and Germany for both omnivores (omnivore, omnivore with a few restrictions, reducetarian, partial vegetarian) and veg*ans (vegetarian and vegan).

To test Hypothesis 1 and 2, we first examined the correlations between our key variables. Given the large number of correlations, we have highlighted correlations that are significant at the *p* < 0.01 level and are of medium size (0.3) or above [58]. The results for Germany are presented above the diagonal, and the results for Australia are presented below the diagonal in Table 4. In Germany and Australia, red meat and poultry consumption had the strongest positive association with Nice and had the strongest negative association with empathy. Fish consumption had the strongest positive association with Necessary and had the strongest negative association with AFP. In both Australia and Germany, intentions to reduce meat consumption had the strongest positive association with PBC and had the strongest negative association with Nice.

To further test Hypothesis 1 and 2, we used multiple regression to determine to what extent speciesism, the 4Ns of the MEMI, empathy, AFP, the Avoidance and Dissociation subscales of the MEJ, and gender predict current red meat, poultry, and fish/shellfish consumption as well as intentions to reduce meat consumption. For intentions to reduce meat consumption, we also used PBC as a predictor. Variables were entered simultaneously, and therefore, only the predictors that incrementally explain variance in meat consumption are significant.

In the Australian sample, the variables predicted 19% of the variance in poultry consumption and 32% of the variance in red meat consumption. Empathy and Nice were the only significant predictors for poultry and red meat. For fish consumption, the variables explained 5% of the variance. AFP was the only significant predictor.

In the German sample, the variables explained 30% of the variance in poultry consumption. AFP, empathy, Necessary, Nice and Dissociation were all unique significant predictors of poultry consumption. The variables explained 25% of variance in meat consumption. Nice and gender were the only two significant predictors for red meat consumption. None of the variables were significant predictors of fish consumption.

For intentions to reduce meat consumption, in the Australian sample, the variables explained 40% of the variance, with empathy, Normal, Natural, Nice and PBC being significant predictors. In the German sample, the variables explained 51% of the variance. Empathy, Necessary, gender and PBC were significant predictors.

The *R*^2^, β, *t*, and *p* statistics for these regressions are presented in Table 5.

To test Hypothesis 3, an independent samples t-test compared men and women on all measures. Due to the large number of comparisons, we applied a Bonferroni correction, dividing the normal significance level of *p* < 0.05 by the number of variables, in this case fourteen, meaning we only focus on comparisons where *p* < 0.004. These comparisons are reported separately for Australia and Germany. The full details of these analyses are presented in Table 6 and Table 7.

In Australia, women scored lower than men on speciesism and Nice, and they scored higher on intentions to reduce meat consumption, AFP, empathy, Avoidance and Dissociation. In Germany, women ate red meat, poultry, and fish on significantly fewer days than men, scored lower on speciesism and all 4Ns, and scored higher on intentions to reduce, AFP, empathy, Avoidance and PBC. For the Australian sample, there were medium effect sizes for Avoidance, speciesism, and empathy, and there was a small effect size for intentions to reduce meat consumption, Dissociation, Nice and AFP. For the German sample, there were large effect sizes for intentions to reduce meat consumption and empathy, medium effect sizes for red meat and poultry consumption, Avoidance, speciesism, AFP, Nice, Natural, and PBC, and a small effect size for Normal, Necessary and fish consumption.

## 4. Discussion

In this study, we investigated socio-cultural variables associated with current and intended meat consumption and whether there was a different pattern in Australian and German samples. Due to the large number of variables, we focus here on those that were significant in the regression, as these variables explained variance above and beyond the other variables in our model. Hypothesis 1 was that meat consumption would be positively associated with speciesism, the four subscales of the MEMI, and the Avoidance and Dissociation subscales of the MEJ. We further predicted it would be negatively associated with empathy towards farmed animals, perceived frequency of common animal farming practices, and identifying as a woman. This was partially supported in both samples. For the regression, Nice and Empathy predicted poultry and red meat consumption in Australia above and beyond the other predictor variables. In Germany, Dissociation, Nice, and gender were predictors of red meat consumption, while Nice, Necessary, Dissociation, empathy, and AFP uniquely predicted poultry consumption. None of the variables were good predictors of fish consumption with AFP being a significant predictor in the Australian regression only.

Hypothesis 2 was that intentions to reduce meat consumption would be negatively associated with speciesism, empathy towards farmed animals, the four subscales of the MEMI, and the Avoidance and Dissociation subscales of the MEJ. We further predicted it would be positively associated with empathy towards farm animals, perceived frequency of common animal farming practices, and perceived behavioural control. This was partially supported in both samples. For the regression in Australia, PBC, empathy, Normal, Natural, and Nice were all predictors of intentions to reduce meat consumption. In the German sample, speciesism, PBC, Necessary, empathy and gender were all predictors of intentions to reduce with women having higher intentions to reduce their meat consumption.

Hypothesis 3 was that relative to men, women would score lower on speciesism, the four subscales of the MEMI, and current meat consumption, and they would score higher on animal farming perceptions, empathy towards farmed animals, and the Avoidance and Dissociation subscales of the MEJ. This was mostly supported in the Australian sample, as relative to men, women scored lower on speciesism, Natural, Normal, Nice, and current frequency of red meat and poultry consumption, and they scored higher on AFP, empathy, Avoidance, Dissociation, and intentions to reduce meat consumption. There were no significant differences in fish consumption or Necessary. In the German sample, relative to men, women scored lower on speciesism, all 4Ns, and current frequency of red meat and poultry consumption, and they scored higher on AFP, empathy, Avoidance, and intentions to reduce meat consumption, but there was no significant difference in Dissociation.

The results of this study are in line with previous findings that those with higher levels of endorsement of the 4Ns reported eating more meat [19] and had lower intentions to reduce their meat intake [59]. Similar to our findings, higher empathy towards animals has also been associated with lower reported levels of meat consumption [34,60]. Our finding that higher levels of PBC were associated with more positive intentions to reduce is also in line with previous research [35,36].

Avoidance was not a significant predictor of any form of meat consumption, and Dissociation only showed a significant positive association with poultry consumption in the German sample. These results slightly contrast with Rothgerber [22] who found a negative relationship between Avoidance and chicken consumption (but not other types of meat), whereas Dissociation was not significantly associated with any form of meat consumption, in his sample of American students.

Higher perceived frequency of animal farming practices was associated with eating poultry on fewer days and having more positive intentions to reduce meat consumption only in Germany. While this measure was created for this study, it was similar to Northrope and Ruby [56], who also found that a higher perceived frequency of animal farming practices was associated with eating red meat, poultry, and fish more frequently in Australia but not in Hong Kong.

One reason that dissociation and AFP were better predictors of poultry consumption in Germany may be due to consumers generally having negative perceptions of welfare conditions for chickens raised for meat with some consumers reporting reducing their meat consumption as a result [47]. This may mean that those who wish to continue eating meat may need to work more actively to dissociate meat from the animal that was killed to produce it.

The lack of relationship of our predictor variables with fish consumption may be due to people thinking about fish differently than other types of meat [22]. It is not immediately clear why the perceived frequency of animal farming practices predicted fish consumption in the Australian sample, given that none of the farming practices items were related to the seafood industry. However, given the increasing concerns about the environmental and animal welfare implications of seafood consumption [61], it may be that those who know more about farmed animal welfare are also more informed about issues related to fishing practices and therefore more likely to reduce their consumption.

Unlike previous research [23], speciesism did not explain any additional variance in our regressions for meat consumption, although it was significantly correlated with meat consumption and predicted intentions to reduce meat consumption only in Germany. This suggests that while by itself higher speciesism is associated with a higher frequency of meat consumption, much of this variance is better explained by the other variables in our model. The same can be said for where the variables were less consistently associated with current and intended meat consumption in our regressions.

The gender differences in our study were consistent with previous findings that relative to men, women scored lower on speciesism and the MEMI and reported eating less meat [21,22,23], and they scored higher on intentions to reduce meat consumption, empathy, Avoidance, and Dissociation [22,28]. Based on our descriptive results, women in Australia ate meat more often than men in Germany and had lower intentions to reduce meat consumption. This was surprising and may reflect how norms around eating meat in Germany have shifted for both men and women. Given that men in both countries ate meat more often and were more resistant to reducing meat consumption in the future, consideration should be given to how best to promote behaviour change. As discussed by Rothgerber [22], the reasons why men may be more resistant to reducing meat consumption may not be due to a lack of information but rather a desire to conform to gender expectations.

### 4.1. Key Finding: People Eat Meat Because They Like the Way It Tastes

One of the key findings from our study was the importance of Nice in predicting current and intended meat consumption. The nicer people thought that meat tastes, the more they ate of it and the less willing they were to reduce their consumption. This suggests that taste is seen as a real barrier to reducing meat consumption, particularly among men, who scored higher on Nice and lower on PBC than women. This is concordant with previous findings that taste was the most common motivation to eat meat in a large-scale German study (*N* = 1807) [62]. Other research supporting the results of our study found that taste is considered one of the biggest barriers to adopting a vegetarian lifestyle particularly among men [63]. Furthermore, participants who more strongly endorsed meat as tasting Nice have reported lower intentions to reduce meat consumption after viewing an animal welfare appeal [59]. Thus, taste seems to be a key factor that needs to be addressed to increase consumers’ willingness to eat less meat.

### 4.2. Strengths and Limitations

The strengths of this study include that it was sufficiently powered to detect small-to-medium effects due to our large, gender-balanced samples sizes in both countries. Germany and Australia are large countries whose meat-eating tendencies have been moving in opposite directions, so examining socio-cultural variables associated with current and intended meat consumption helps us to better understand these trends. Furthermore, by pre-registering and sharing our materials and data on the OSF, this study advances the cause of open science. However, there are also limitations to mention. Intentions to reduce meat consumption may not reflect actual behavioural intentions but rather a desire by participants to present themselves positively. This may particularly be the case when intentions are initially driven by emotional reactions, such as empathy towards the suffering of animals, but behaviour is driven by visceral impulses, such as hunger [64]. As this study was correlational by design, it is not possible to draw any conclusions about cause and effect, and it is not clear to what extent these samples are representative of the broader population, limiting the generalisability of our results. Our results regarding Avoidance in Germany, and AFP in general, should also be interpreted with caution giving the lower internal reliability for these measures.

### 4.3. Practical Implications

Given that the two conflicting motives of taste and empathy were the most strongly related to current frequency of meat consumption, interventions for behaviour change may work best by focusing on these factors. Overcoming the hedonic desire for meat consumption is likely complex and may be best targeted from many angles. For example, one could have people confront the meat paradox by reminding them that an animal was killed to produce their meal. Previous research found that just reminding participants that meat came from an animal was enough to reduce anticipated liking [53,65,66]. Given the generally high levels of animal welfare concern in Germany, an emphasis on the suffering of animals in the meat industry may be a useful motivator for reducing meat intake. Research in Australia found that people tend to be sceptical of information shared by animal activists online [66], which may limit the impact information gained this way may have on the general population. For Australian consumers, the most trusted source of livestock animal welfare information was product labels and information shared by family and friends [46]. Labelling on meat may be an effective way to provide consumers with information about the welfare standards and environmental impact of the meat they buy and nudge them into making better decisions.

### 4.4. Future Directions

The challenge here is how best to draw the connection of meat to the animal that was killed to produce it without triggering other dissonance-reducing strategies, such as denying animals the ability to suffer [67]. One study found that reactance was reduced when health concerns were presented as “surprising food facts”, which led to reduced willingness to eat a beef burger [68]. Other interventions could encourage consumers to try tasty vegetarian alternatives [69] and increase perceived behavioural control to reduce meat consumption, such as through increased familiarity with vegetarian meals through initiatives such as Meatless Mondays [70]. Future research should also investigate the influences of meat consumption in different cultural contexts, particularly as countries vary considerably in their consumption rates and exposure to animal processing for meat consumption [65,66].

## 5. Conclusions

This study demonstrates that taste and empathy are two important and conflicting factors linked to people’s current and intended meat consumption. It supports previous research that men consume more meat than women and are more resistant to reducing meat consumption in the future. It also highlights how attitudes in Germany related to meat consumption might differ from Australia with participants in Germany overall having more positive attitudes towards reducing their meat consumption. This may be due to consumers in Germany having more negative perceptions of animal welfare practices, particularly for poultry. Interventions that focus on increasing empathy for farmed animals and addressing concerns about taste may help promote decreased meat consumption in both Australia and Germany.

## Figures and Tables

**Table 1:** Dietary group membership for participants from Australia and Germany.

Dietary Group	Australia		Germany	
	*n*	%	*n*	%
Omnivore	252	63.2	140	35.1
Omnivore with a few restrictions	42	10.6	43	10.8
Reducetarian/Partial vegetarian	66	16.6	135	33.8
Vegetarian	27	6.8	59	14.8
Vegan	11	2.8	22	5.5
Missing	1	0.3	-	-

**Table 2:** Perceived frequency of animal farming practices in Australia and Germany.

Item	Australia *M* (*SD*)	Germany *M* (*SD*)
1. Pigs are provided pain relief when undergoing painful procedures such as castration. (R)	26.53 (27.91)	29.70 (28.38)
2. Overcrowding of pigs in sheds lead to stress-induced behaviours such as cannibalism.	51.60 (28.26)	67.74 (26.08)
3. Transport of livestock over long distances cause distress and injury to the animals.	71.62 (24.92)	81.59 (19.97)
4. Livestock animals are protected from seeing other animals killed in slaughterhouses. (R)	28.67 (27.87)	26.23 (26.38)
5. Free-range chickens spend time in outside spaces (e.g., a yard or pasture). (R)	58.06 (27.40)	47.84 (26.89)
6. Chickens raised for meat get ammonia burns on their feet due to continued exposure to their own waste.	51.98 (27.52)	61.39 (25.08)

Note. Responses are on a sliding scale from 0% of the time to 100% of the time. R = reverse-scored item.

**Table 3:** Means and standard deviations for measures in Australia and Germany.

Measure	Australia		Germany	
	Omnivores *n* = 360 *M* (*SD*)	Veg*ans *n* = 38 *M* (*SD*)	Omnivores *n* = 318 *M* (*SD*)	Veg*ans *n* = 81 *M* (*SD*)
Poultry Intake	4.03 (1.51)	1.03 (0.16)	3.04 (1.40)	1.00 (0.00)
Red Meat Intake	3.29 (1.37)	1.00 (0.00)	2.58 (1.32)	1.00 (0.00)
Fish Intake	2.27 (1.11)	1.05 (0.32)	2.03 (0.94)	1.19 (0.50)
Intentions to Reduce	2.45 (1.34)	5.66 (2.49)	3.37 (1.61)	6.14 (2.10)
Speciesism	3.06 (1.20)	1.80 (0.74)	2.93 (1.13)	1.77 (0.77)
AFP	58.36 (15.11)	78.93 (15.90)	64.68 (15.17)	80.20 (11.00)
Empathy	3.51 (1.60)	6.19 (1.11)	3.51 (1.48)	6.12 (1.09)
Necessary	4.81 (1.26)	3.46 (1.63)	4.05 (1.51)	2.60 (1.39)
Normal	2.63 (1.24)	2.28 (1.59)	2.22 (1.24)	1.80 (1.10)
Natural	3.60 (1.54)	2.00 (1.30)	3.08 (1.57)	1.62 (0.88)
Nice	5.05 (1.43)	2.81 (1.70)	4.81 (1.29)	2.67 (1.55)
Avoidance	4.95 (1.44)	5.31 (1.03)	4.38 (1.37)	4.16 (1.31)
Dissociation	4.82 (1.55)	3.84 (1.73)	4.55 (1.45)	2.69 (1.59)
PBC	4.64 (1.25)	6.49 (1.25)	4.99 (1.13)	6.74 (7.14)

Note. AFP refers to animal farming perceptions. PBC refers to perceived behavioural control.

**Table 4:** Pearson’s correlations of meat consumption and intentions to reduce meat consumption with speciesism, the four subscales of the Motivations to Eat Meat Inventory (MEMI), perceived behavioural control (PBC), empathy, animal farming perceptions (AFP), and meat-eating justification (MEJ) avoidance and dissociation.

Measure	1	2	3	4	5	6	7	8	9	10	11	12	13	14
1. Poultry Intake	−	0.43 *	0.28 *	−0.46 *	0.31 *	−0.32 *	−0.40 *	0.38 *	0.14 *	0.29 *	0.42 *	0.07	0.32 *	−0.55 *
2. Red Meat Intake	0.41 *	-	0.19 *	−0.44 *	0.28 *	−0.26 *	−0.37 *	0.31 *	0.10	0.28 *	0.42 *	−0.03	0.20 *	−0.47 *
3. Fish Intake	0.18 *	0.17 *	-	−0.20 *	0.17 *	−0.14 *	−0.22 **	0.23 *	0.11	0.21 *	0.18 *	0.02	0.16 *	−0.32 *
4. Intentions to Reduce	−0.45 *	−0.50 *	−0.08	-	−0.50 *	0.37 *	0.57 *	−0.43 *	−0.19 *	−0.45 *	−0.58 *	0.07	−0.22 *	0.63 *
5. Speciesism	0.27 *	0.32 *	0.05	−0.43 *	-	−0.44 *	−0.63 *	0.40 *	0.25 *	0.42 *	0.45 *	−0.13	0.14 *	−0.39 *
6. AFP	−0.24 *	−0.23 *	−0.15 *	0.37 *	−0.40 *	-	0.36 *	−0.40 *	−0.26 *	−0.33 *	−0.31 *	−0.07	−0.21 *	0.39 *
7. Empathy	−0.34 *	−0.40 *	−0.06	0.57 *	−0.57 *	0.33 *	-	−0.34 *	−0.13 *	−0.38 *	−0.53 *	−0.07	−0.21 *	0.41 *
8. Necessary	0.23 *	0.36 *	0.16 *	−0.28 *	0.22 *	−0.20 *	−0.28 *	-	0.39 *	0.73 *	0.50 *	0.05	0.26 *	−0.41 *
9. Normal	0.12	0.09	0.02	−0.02	0.17 *	−0.18 *	−0.05	0.16 *	-	0.53 *	0.28 *	0.04	0.14 *	−0.25 *
10. Natural	0.25 *	0.34 *	0.15 *	−0.34 *	0.33 *	−0.24 *	−0.32 *	0.61 *	0.42 *	-	0.44 *	0.01	0.22 *	−0.41 *
11. Nice	0.35 *	0.51 *	0.08	−0.42 *	0.35 *	−0.24 *	−0.43 *	0.50 *	0.23 *	0.43 *	-	−0.02	0.25 *	−0.42 *
12. Avoidance	−0.04	−0.09	0.03	0.22 *	−0.33 *	0.12	0.35 *	0.02	−0.02	−0.13	−0.11	-	0.64 *	−0.10
13. Dissociation	0.06	0.04	0.08	0.02	−0.17 *	−0.05	0.14 *	0.14 *	0.01	−0.02	0.10	0.73 *	-	−0.32 *
14. PBC	−0.37 *	−0.54 *	−0.13	0.57 *	−0.38 *	0.33 *	0.39 *	−0.35 *	−0.17 *	−0.40 *	−0.41 *	0.12	−0.01	-

Note. * Correlation is significant at the *p* < 0.01 level. Lighter shades of orange indicate medium positive correlations (*r* ≥ 0.3), darker shades of orange indicate large positive correlations (*r* ≥ 0.5). Lighter shades of blue indicate medium negative correlations, darker shades of blue indicate large positive correlations. Results for Germany are presented above the diagonal line; results for Australia are presented below the diagonal line.

**Table 5:** Regression summary table of predictor variables on current meat consumption and intentions to reduce meat consumption in Australia and Germany.

	Australian Sample				German Sample			
Predictor	*R* ^2^	β	*t*	*p*	*R* ^2^	β	*t*	*p*
Poultry	0.19				0.30			
Speciesism		0.04	0.72	0.471		−0.02	−0.25	0.806
AFP		−0.09	−1.81	0.071		−0.10	−2.02	**0.043**
Empathy		−0.20	−3.27	**0.001**		−0.16	2.56	**0.011**
Necessary		−0.01	−0.11	0.909		0.21	3.08	**0.002**
Normal		0.01	0.16	0.876		−0.04	0.70	0.487
Natural		0.07	1.07	0.284		−0.09	−1.12	0.264
Nice		0.20	3.41	**<0.001**		0.18	3.20	**0.001**
Avoidance		0.07	0.94	0.347		−0.03	−0.41	0.683
Dissociation		0.02	0.34	0.733		0.20	3.13	**0.002**
Gender		0.00	0.09	0.931		0.08	1.50	0.134
Red Meat	0.32				0.25			
Speciesism		0.06	1.00	0.317		−0.03	−0.48	0.629
AFP		−0.05	−1.05	0.296		−0.07	−1.39	0.167
Empathy		−0.16	−2.84	**0.005**		−0.11	−1.65	0.100
Necessary		0.06	1.01	0.310		0.06	1.32	0.187
Normal		−0.07	−1.54	0.125		−0.09	−1.62	0.106
Natural		0.11	1.72	0.086		0.03	0.336	0.737
Nice		0.35	6.41	**<0.001**		0.24	4.07	**<0.001**
Avoidance		0.04	0.62	0.537		−0.05	−0.81	0.418
Dissociation		0.01	0.17	0.869		0.12	1.86	0.063
Gender		0.03	0.66	0.509		0.15	2.93	**0.004**
Fish	0.05				0.09			
Speciesism		−0.03	−0.47	0.639		0.00	0.02	0.983
AFP		−0.14	−2.44	**0.015**		0.00	0.03	0.977
Empathy		0.02	0.25	0.803		−0.12	−1.69	0.092
Necessary		0.10	1.43	0.155		0.14	1.83	0.068
Normal		−0.06	−1.02	0.310		0.01	0.18	0.858
Natural		0.10	1.43	0.152		0.01	0.16	0.876
Nice		−0.03	−0.50	0.619		−0.00	−0.04	0.965
Avoidance		0.01	0.11	0.911		−0.01	−0.08	0.936
Dissociation		0.01	0.74	0.461		0.09	1.24	0.216
Gender		0.05	0.85	0.397		0.06	0.97	0.334
Intentions to Reduce	0.40				0.51			
Speciesism		−0.03	−0.48	0.632		−0.12	−2.31	0.022
AFP		0.05	1.13	0.258		−0.00	0.035	0.972
Empathy		0.27	5.26	**<0.001**		0.27	5.30	**<0.001**
Necessary		−0.02	−.38	0.705		−0.12	−2.05	**0.041**
Normal		0.11	2.42	**0.016**		0.07	1.46	0.149
Natural		−0.12	−2.07	**0.039**		−0.07	−1.15	0.253
Nice		−0.14	−2.78	**0.006**		−0.04	−0.81	0.416
Avoidance		0.03	0.38	0.702		0.09	1.30	0.194
Dissociation		0.04	0.51	0.611		−0.03	−0.52	0.606
Gender		−0.05	−1.18	0.237		−0.10	−2.18	**0.030**
PBC		0.29	6.18	**<0.001**		0.39	8.72	**<0.001**

Note. AFP refers to animal farming perceptions. PBC refers to perceived behavioural control over future meat consumption. *p* values < 0.05 are indicated in bold.

**Table 6:** Differences by gender in Australia for all outcome measures.

	Men	Women					
	*M* (*SD*)	*M* (*SD*)	*M_diff_* Men–Women	*df*	*t*	*p*	*d*
Red meat	3.28 (1.42)	2.86 (1.45)	−0.42	391	−2.92	0.004	0.29
Poultry	3.92 (1.60)	3.57 (1.74)	−0.35	391	−2.07	0.039	0.21
Fish	2.21 (1.03)	2.11 (1.20)	−0.10	391	−0.87	0.387	0.09
Intentions	2.22 (1.33)	2.71 (1.30)	0.49	391	3.53	**<0.001**	0.37
Speciesism	3.29 (1.29)	2.62 (1.04)	−0.68	373.24 *	−5.72	**<0.001**	0.58
AFP	57.58 (15.10)	62.67 (16.83)	5.09	386.88 *	3.16	**0.002**	0.32
Empathy	3.29 (1.78)	4.20 (1.57)	0.90	384.37 *	5.32	**<0.001**	0.54
Necessary	4.68 (1.38)	4.67 (1.31)	−0.01	391	−0.05	0.962	0.01
Normal	2.72 (1.31)	2.44 (1.24)	−0.28	391	−2.15	0.032	0.22
Natural	3.65 (1.64)	3.24 (1.52)	−0.41	391	−2.60	0.010	0.26
Nice	5.11 (1.56)	4.58 (1.58)	−0.53	391	−3.38	**<0.001**	0.34
Avoidance	4.57 (1.50)	5.37 (1.20)	0.80	372.32 *	5.86	**<0.001**	0.59
Dissociation	4.44 (1.60)	5.02 (1.54)	0.58	391	3.66	**<0.001**	0.37
PBC	4.70 (1.31)	4.93 (1.39)	0.23	391	1.66	0.097	0.17

Note. Equal variances not assumed are marked *. AFP refers to animal farming perceptions and PBC refers to perceived behavioural control. *p*-values significant at the Bonferroni corrected level of <0.004 indicated in bold.

**Table 7:** Differences by gender in Germany for all outcome measures.

	Men	Women					
	*M* (*SD*)	*M* (*SD*)	*M_diff_* Men–Women	*df*	*t*	*p*	*d*
Red meat	2.69 (1.47)	1.85 (1.03)	−0.83	356.19 *	−6.52	**<0.001**	0.66
Poultry	3.01 (1.44)	2.26 (1.46)	−0.75	389	−5.12	**<0.001**	0.52
Fish	2.00 (0.94)	1.72 (0.90)	−0.28	389	−2.97	**0.003**	0.30
Intentions	2.98 (1.48)	3.99 (1.60)	1.01	312	5.77	**<0.001**	0.66
Speciesism	3.07 (1.15)	2.30 (1.06)	−0.77	389	−6.91	**<0.001**	0.70
AFP	63.37 (15.29)	72.04 (14.88)	8.67	389	5.68	**<0.001**	0.58
Empathy	3.29 (1.46)	4.79 (1.70)	1.49	375.74 *	9.26	**<0.001**	0.94
Necessary	4.10 (1.50)	3.44 (1.61)	−0.66	389	−4.23	**<0.001**	0.43
Normal	2.39 (1.32)	1.90 (1.08)	−0.49	379.35 *	−4.02	**<0.001**	0.41
Natural	3.29 (1.62)	2.31 (1.36)	−0.98	382.20 *	−6.50	**<0.001**	0.66
Nice	4.88 (1.31)	3.91 (1.68)	−0.97	360.67 *	−6.36	**<0.001**	0.65
Avoidance	4.00 (1.37)	4.68 (1.27)	0.69	389	5.16	**<0.001**	0.52
Dissociation	4.25 (1.58)	4.12 (1.76)	−0.13	389	−0.78	0.436	0.08
PBC	4.98 (1.14)	5.74 (1.26)	0.76	382.19 *	6.23	**<0.001**	0.63

Note. Equal variances not assumed marked *. AFP refers to animal farming perceptions and PBC refers to perceived behavioural control. *p*-values significant at the Bonferroni corrected level of <0.004 indicated in bold.

## Data Availability

Anonymized data files are available via the OSF at https://osf.io/j8vtp/, accessed on 28 November 2023.
